# Notes and Formulæ

**Published:** 1889-08

**Authors:** 


### PRACTICAL NOTES AND FORMULÆ.

---

**Treatment of Granular Lids.**—Dr. F. M. Scott writes the Kansas Med. Index as follows: I have had good success in treating granulated lids by brushing the conjunctiva's surface three or four times a week with a solution of nitrate of silver, five grains to an ounce of water, and immediately washing it off with a weak solution of salt and water; and having the patient instil into the eyes, once a day, five drops of the following:

| | |
|---|---|
| Boracic acid, | gr. xv. |
| Tannic acid, | gr. x. |
| Glycerin, | ʒ iss. |
| Rose water, q. s. ad. | ℥ j. |

Two cases were quite bad and of long duration, having considerable degree of pannus. I like the weaker solutions of silver better than to risk those stronger than five grains to the ounce.—*Ex.*

**Anodyne Liniment.**—As an anodyne liniment, the following is recommended by Dr. Brubaker: ℞ Chlor. hydr., ʒij, liniment saponis, f ℥ iv. M. Sig. Rub on the affected part.

**Local Counter Irritant for Children.**—

| | | |
|---|---|---|
| ℞ | Pul. cinnamon, | ℥ i. |
| | Pul. allspice, | ʒ ii. |
| | Pul. black pepper, | ʒ ii. |
| | White of two eggs. | |

M. Sig. Apply over the stomach and abdomen.—*Med. Waif.*

**Catarrhal Diarrhœa of Children.**—

| | | |
|---|---|---|
| ℞ | Syr. rhei aromat., | ʒ v. |
| | Mist. cretæ prep., | ℥ iss. |
| | Syr. acaciæ, | q. s. ad. ℥ iii. |

M. Et. Sig. Teaspoonful every two hours. In simple catarrhal diarrhœa due to dentition, do not lock the bowels up, but restrict the diet.

If the child is nervous, it may have convulsions. If the child have convulsions place it in a hot mustard bath and let the mother hold the child until her hands begin to tingle. Give an enema of one grain of hydrate of chloral and twenty grains of bromide of potassium, dissolved in ℥ ii of water. After child has rested give broken doses of calomel.—Dr. Starr, in *Medical Waif.*

**Antifermenting Powder.**—Dr. Dujardin-Beaumets frequently prescribes the following powders in cases of dilatation of the stomach and in all such cases in which it is desirable to combat secondary fermentations which occur in the stomach and intestines:

℞ Bismuthi salicylat.
Magnesiæ carbonat.
Natri bicarbonat ää ʒ ijss
M. Ft. Chart. No. 30.
Sig. One powder at breakfast and at dinner.

Or, ℞ Bismuthi salicylat.
Naphthol.
Magnesiæ carbonat ää ʒ ijss
M. Ft. Chart. No. 30.
Sig. One powder at breakfast and dinner.

We have had most excellent results in anal and vulvar pruritus, and indeed in pruritus of all descriptions with the following:

℞ Campho-phenique ʒ i
Lanolin ʒ i—ʒ iss
M. Apply twice daily after washing with campho-phenique (or in its absence plain castile) soap and thoroughly drying the surface.

Itching is subdued at once, and in ordinary cases, depending on local causes, a cure is rapidly effected.

As a covering for small wounds, Prof. Forbes uses at the Jefferson Clinic:

℞ Olei ricini ʒ iv.

Collodii ℥ i.

Hydronapthol 10%

—*The College and Clinical Record.*

**Antiseptic Mixture.**—To avoid the toxic properties of carbolic acid and corrosive sublimate when used in disinfectant solution, Dr. E. Rotter has combined a large number of antiseptics in small proportions to form the following mixture, which acts as a serviceable antiseptic and is free from poisonous properties:

| | |
|---|---|
| ℞ Chloride of soda | 0.25 |
| Chloride of zinc | |
| Sulphocarbolate of zinc | ää 5.0 |
| Boracic acid | 3.0 |
| Salicylic acid | 0.6 |
| Thymol | 0.1 |
| Citric acid | |

This powder when dissolved in one litre of water gives a perfectly clear and efficient antiseptic solution. It is more powerful than the ordinary solutions of corrosive sublimate and carbolic acid. The author has successfully used this mixture in surgical and obstetrical practice.—*Centralblatt fur Chirurgie*, No. 3, 1889. [P. J. R.]

